# Melatonin synergizes BRAF-targeting agent vemurafenib in melanoma treatment by inhibiting iNOS/hTERT signaling and cancer-stem cell traits

**DOI:** 10.1186/s13046-019-1036-z

**Published:** 2019-02-04

**Authors:** Jiaojiao Hao, Wenhua Fan, Yizhuo Li, Ranran Tang, Chunfang Tian, Qian Yang, Tianhua Zhu, Chaoliang Diao, Sheng Hu, Manyu Chen, Ping Guo, Qian Long, Changlin Zhang, Ge Qin, Wendan Yu, Miao Chen, Liren Li, Lijun Qin, Jingshu Wang, Xiuping Zhang, Yandong Ren, Penghui Zhou, Lijuan Zou, Kui Jiang, Wei Guo, Wuguo Deng

**Affiliations:** 1 Institute of Cancer Stem Cells and The Second Affiliated Hospital, Dalian Medical University, Dalian, China; 2 State Key Laboratory of Oncology in South China; Collaborative Innovation Center of Cancer Medicine, Sun Yat-sen University Cancer Centre, Guangzhou, China; 3 Nanjing Maternity and Child Health Care Hospital, Women’s Hospital of Nanjing Medical University, Nanjing, China; 4 Sun Yat-sen Memorial Hospital of Sun Yat-sen University, Guangzhou, China; 5 Cloud Health Genomics Ltd, Shanghai, China

**Keywords:** Melatonin, Vemurafenib, NF-κB, iNOS, hTERT, Cancer stem cell

## Abstract

**Background:**

As the selective inhibitor of BRAF kinase, vemurafenib exhibits effective antitumor activities in patients with V600 BRAF mutant melanomas. However, acquired drug resistance invariably develops after its initial treatment.

**Methods:**

Immunohistochemical staining was performed to detect the expression of iNOS and hTERT, p-p65, Epcam, CD44, PCNA in mice with melanoma xenografts. The proliferation and migration of melanoma cells were detected by MTT, tumorsphere culture, cell cycle, cell apoptosis, AO/EB assay and colony formation, transwell assay and scratch assay in vitro, and tumor growth differences were observed in xenograft nude mice. Changes in the expression of key molecules in the iNOS/hTERT signaling pathways were detected by western blot. Nucleus-cytoplasm separation, and immunofluorescence analyses were conducted to explore the location of p50/p65 in melanoma cell lines. Flow cytometry assay were performed to determine the expression of CD44. Pull down assay and ChIP assay were performed to detect the binding ability of p65 at iNOS and hTERT promoters. Additionally, hTERT promoter-driven luciferase plasmids were transfected in to melanoma cells with indicated treatment to determine luciferase activity of hTERT.

**Results:**

Melatonin significantly and synergistically enhanced vemurafenib-mediated inhibitions of proliferation, colony formation, migration and invasion and promoted vemurafenib-induced apoptosis, cell cycle arresting and stemness weakening in melanoma cells. Further mechanism study revealed that melatonin enhanced the antitumor effect of vemurafenib by abrogating nucleus translocation of NF-κB p50/p65 and their binding at iNOS and hTERT promoters, thereby suppressing the expression of iNOS and hTERT. The elevated anti-tumor capacity of vemurafenib upon co-treatment with melatonin was also evaluated and confirmed in mice with melanoma xenografts.

**Conclusions:**

Collectively, our results demonstrate melatonin synergizes the antitumor effect of vemurafenib in human melanoma by inhibiting cell proliferation and cancer-stem cell traits via targeting NF-κB/iNOS/hTERT signaling pathway, and suggest the potential of melatonin in antagonizing the toxicity of vemurafenib and augmenting its sensitivities in melanoma treatment.

**Electronic supplementary material:**

The online version of this article (10.1186/s13046-019-1036-z) contains supplementary material, which is available to authorized users.

## Introduction

Melanoma is one of the most threatening malignancies and has high metastatic potential. Although in the recent years, significant progresses have been made in melanoma treatment with the appearance and widespread application of the combinational immunotherapy [1–4], it is still necessary to explore other treatment options to get better clinical output because the response rates to immunotherapy are not 100%. This might be mainly due to that the antigens selected for these approaches do not cover the full spectrum of melanoma cells present in a tumor [5, 6]. The studies on cancer stem cells in melanoma raise the possibility that this long-lived tumor subpopulation is resistant to clinical therapy [7]. Normal stem cells are thought to achieve their longevity by several mechanisms among which are slow divisions, anti-apoptotic mechanisms, and expression of efflux pumps that provide protection from toxins [7, 8], and the design of more effective therapeutic strategies targeting melanoma stem cells and associated molecular pathways and their application hold promise for melanoma treatment. Inflammation is an important feature of the tumor microenvironment in melanoma, and previous studies showed that inducible nitric oxide synthase (INOS), one of the most common inflammation factors, is an important inducer of melanoma tumorigenesis, tumor growth, invasion and metastasis [9, 10], and INOS abrogation has been proved to contribute to melanoma treatment.

BRAF mutations have been found in melanoma [11, 12], and V600E is the most common mutation in BRAF leading to constitutive activation of the MAPK signaling pathway in malignant melanomas [13]. The MAPK signaling pathway is involved in activation of BRAF which phosphorylates and activates MEK, and in turn phosphorylates and activates ERK [14]. These reactions result in the activation of transcription factors that regulate cell survival, proliferation and differentiation. Vemurafenib, a small molecule inhibitor of serine/threonine protein kinase BRAF, shows initial good clinical responses [15]. Unfortunately, the relative initial success of vemurafenib has been dampened by the development of acquired resistance to the drug [16]. Moreover, the patients received vemurafenib treatment easily present a severe anterior uveitis secondary to this drug. In general, the reactivation of MAPK signaling pathway, the bypass of oncogenic pathway via activation of alternative signaling pathways, and other uncharacterized mechanisms are considered to be the cause of therapeutic resistance in kinase-driven cancers [17–19].

Melatonin (N-acetyl-5-methoxytryptamine) is a ubiquitous physiological mediator secreted by the pineal gland. In mammals, the pineal gland [20, 21] secretes melatonin into the blood circulation to exert a range of well-documented physiological functions [22]. It is well-known melatonin is an important endogenous synchronizer of the circadian day–night rhythm and seasonal biorhythms on a variety of target organs [23–25]. Functionally, melatonin has been widely documented because of its significant antitumor effects on the ovarian carcinoma [26], human melanoma [27] and breast cancer [28] et.al. In addition, melatonin can induce cancer cell apoptosis and suppress tumor metastasis, angiogenesis and inflammatory reaction, which indicate its potential clinical applications [29–31]. Melatonin has been shown to function as a potent combination therapeutic agent in human cancer cells by enhancing the efficacy of conventional anticancer agents and meanwhile reducing their side effects [32–34]. Notably, melatonin reduces cancer cell proliferation and decreases self-renewal and clonogenic capability through the decreased expression of stem cell markers [35]. However, the mechanisms associated with the melatonin-regulated gene expression remain unclear. Therefore, it is important to clarify the underlying molecular mechanisms involved in the combination and to discover newly potential therapeutic targets.

In this study, we assessed the role of melatonin in the enhancement of the vemurafenib-mediated antitumor effect and identified the underlying mechanism of the combination treatment in melanoma.

## Methods

### Cell lines and cell culture

Human melanoma cell lines SK-Mel-28, A375, A431 and G361 were all obtained from the American Type Culture Collection (ATCC). All the cells were grown in Dulbecco^,^s Modified Eagle Medium (HyClone, Thermo Scientific) supplemented with 10% heat-inactivated fetal bovine serum (Gibco).

### Establishment of melanoma cell line with relative vemurafenib resistances

Briefly, we cultivated A375 cells under the treatment of increasing amounts of vemurafenib (VE), firstly 0.5 μM VE was used for 2 weeks, and then 1μΜ VE was added as a part of the culture medium. Cells that survive the conditions were selected and amplified.

### Western blot

Proteins from melanoma cell lysate were quantified using a BCA protein assay kit and were loaded onto a 10% polyacrylamide gel (SDS-PAGE), then then transferred onto a polyvinylidene fluoride (PVDF, Millipore, USA) membrane. Western blots were incubated with the specific primary antibodies. Finally immunoreactivity were detected by enhanced chemiluminescence.

### Reagents and antibodies

Melatonin was purchased from J&K, Chemical Ltd. Vemurafenib was obtained from Selleck (PLX4032) and dissolved in dimethyl sulphoxide (DMSO) before addition to the complete cell culture medium. For the experiment, the solution of melatonin (1 M) and vemurafenib (VE) (10 mM) in DMSO was prepared and kept at 4 °C for further dilution in culture medium to maintain stability of used drugs. The InSolution™ NF-κB Activation Inhibitor controlling the biological activity of NF-κB (481407) was bought from Merck Millipore. Antibodies against β-catenin, MMP-1, MMP-9, Apaf-1 were purchased from Santa Cruz (USA). The antibodies against β-actin, IKKα, IKKβ, p-IKKα/β, IκB-α, p-IκB-α, cleaved caspase-3, cleaved caspase-9, cleaved PARP, Bcl-2, NF-κB p50, p65, p-PDK1, p-PTEN, p-AKT and AKT were purchased from Cell Signaling Technology (USA). The anti-TFIIB, E-cadherin antibodies were purchased from Proteintech group (USA), and anti-iNOS antibody was purchased from Wanlei Biotechnology (China). The anti-BRAF V600E antibody was purchased from Omnimabs (USA).

### Plasmid vectors

The transfection was performed using Lipofectamine 3000 reagent (Invitrogen, Carlsbad, CA). Recombinant plasmid vectors pGL3-hTERT-438 expressing luciferase driven by hTERT promoter (− 378 to + 60) were produced in our lab.

### Tumorsphere culture

Cells with indicated treatment were digested into single cell with trypsin-EDTA and were respectively seeded in 35 mm non-treated cell culture dishes (BIOFIL, 2000 cells/dish) with continuous culture in DMEM/F12 medium (HyClone) containing B27 supplement (Gibco), N_2_ supplement (Gibco), bFGF (20 ng/ml), and EGF (20 ng/ml) for two weeks. Then the pictures of the formed tumorspheres were taken by inverted microscope (Leica) and the number of the spheres with diameter larger than 50um was counted.

### Flow cytometry assay of CD44

Expression of stemness-associated marker, CD44, was detected by flow cytometer. A375 and SK-MEL-28 cells with indicated treatment were digested with trypsin-EDTA and washed twice in PBS containing 2% BSA and centrifuged at 300×g for 3 min. Cells were divided into two groups and resuspended in 100 ul PBS with 2% BSA on ice. Then the antibody APC-IgG and APC-CD44 (BD Pharmingen) were respectively added into single tube of each group on ice to incubate for 30 min. The fluorescence value was detected finally by FACS Accuri C6 (Genetimes Technology Inc.).

### Cell viability assay

Cell viability was assessed using the MTT assay (Roche Diagnosis, Indianapolis, IN). Briefly, melanoma cell lines were seeded onto 96 well plates for following overnight incubation. Five replicated wells were put up for each group. Then cells were treated with melatonin or vemurafenib by indicated dose. Finally, the effect for cell viability was assessed by the absorbance of the supernatant at a wavelength of 450 nm comparing to the vehicle-treated control group. The drug concentration required to cause 50% cell growth inhibition (IC_50_) was determined by interpolation from dose-response curves.

### Colony formation assay

Melanoma cells were seeded into six well plate (2 × 10^3^ per well) and were incubated for 12 h. Then, the medium was removed and cells were exposed to various drugs. After 24 h, cells were changed into fresh medium containing 10% FBS and incubated in a 37 °C, 5% CO_2_ incubator for 14 days until cells grew into macroscopic colonies. Finally, the medium was removed, and the colonies were stained by 0.1% crystal violet and counted.

### Scratch assay

A375 and SK-mel-28 cells treated with indicated doses of vemurafenib or melatonin. The cells were seeded into six-well plates and incubated in the medium with 2.5% FBS until grown to full confluency, then scraped by a sterile 200 μl pipette tip. After 36 h, medium was replaced with PBS buffer and the wound gap was photographed by inverted microscope (Leica DM 14000B microscope fixed with digital camera) at 0 h and 48.

### Transwell assay

For the transwell assay, 4 × 10^4^ cells were treated with indicated doses of vemurafenib or melatonin per chamber were plated. Cells were allowed to invade through the matrigel-coated inserts. After the cells that remained in the gel or attached to the upper side of the filter were removed with cotton swabs, the invaded cells were stained with 0.1% crystal violet solution and then was photographed using a Leica DM 14000B microscope fixed with digital camera. Finally, we used image-Pro plus software for counting the invaded cells. The concrete steps are as following: First, load the file to be analyzed from the folder, and then select the rectangular AOI tool, define the AOI to cover the image, and click on select colors in the Count/Size dialog box, and select Histogram Based button to set the range. Next, from the Measure menu in the Count/Size dialog box, select the Select Measurements command. Add the Area measurement in the Filter Ranges, and set the threshold, then click on Measure, and click the count in the Count/Size dialog box. From the View menu in the Count/Size dialog box, select the Statistics command.

### Cell cycle and apoptosis assay

In brief, A375 and SK-mel-28 cells (10^5^ cells) seeded in 6-well plates were treated with indicated doses of vemurafenib or melatonin. Cell cycle assay: after 48 h, cells were collected and stained DNA with PI, finally sorted by FACS Accuri C6 (Genetimes Technology Inc.) and analyzed by using FlowJo 7.6 software; Apoptosis assay: after 48 h, cells were collected subsequently stained simultaneously with FITC-labeled annexin V and PI. Stained cells were analyzed using FACS Accuri C6 (Genetimes Technology Inc.).

### Acridine orange /ethidium bromide fluorescence staining

A375 or SK-mel-28 cells were grown on chamber slides and treated with indicated doses of vemurafenib or melatonin. After 48 h, cells were washed by PBS, and then fixed by 95% ethanol for 15 min. After slightly drying cells, 5 ul AO/EB (50 μg/ml) were added with gently pipetting to mix before photographing by Leica DM 14000B microscope fitted with digital camera.

### Pulldown assay

The biotin-labeled double-stranded oligonucleotide probes, which correspond to hTERT promoter sequence or iNOS promoter sequence, were synthesized by PCR using biotin-labeled primers from TAKARA Company. The nucleus proteins (400 μg) were mixed with double-strand biotinylated hTERT or iNOS promoter probe (4 μg), streptavidin agarose beads (50 ml) in 500 ml PBSI buffer (0.5 mM PMSF, 10 mM NaF, 25 mM β-glycerophosphate) and rotated for 4 h at RT. The beads were centrifuged, washed with PBSI buffer for two times, and then were resuspended by loading buffer and boiled at 100 °C for 10 min. The supernatant was analyzed by Western blot.

### Chromatin immunoprecipitation assay (ChIP)

Briefly, the A375 and SK-mel-28 cells were fixed with 1% formaldehyde, sonicated on ice to shear the DNA into the fragments from 200 bp to 500 bp. The lysate were subjected to immunoprecipitations with anti-p65 or non-specific rabbit IgG. The immunoprecipitated DNA was subjected to PCR to amplify a 220 bp fragment of hTERT promoter or a 510 bp fragment of iNOS promoter. The PCR products were run electrophoretically on a 1% agarose gel and visualized by ethidium bromide staining.

### Confocal immunofluorescence assay

A375 and SK-mel-28 cells were incubated on chamber slides in 6-well plates with indicated treatment, cells fixed for 10 min at room temperature (RT) with 4% paraformaldehyde, and then permeabilized with PBST (PBS with 0.2%Triton X-100), blocked with bovine serum albumin (BSA) 30 min and incubated with Cytochrome-c, or p65 or p50 antibodies (1:200 dilution) for overnight at 4 °C. Following 10-min washes for three times with PBS, cells were incubated with the fluorescein isothiocyanate- and rhodamineconjugated secondary antibodies for 30 min. Subsequently, the nuclei of stained samples were mounted with Vectashield solution containing 4′6-diamidino-2-phenylindole (DAPI). After five additional 10-min washes, the results were visualized by Leica DM 14000B confocal laser scanning microscope.

### Xenograft tumor models of human melanoma

Male nude mice (4-5 weeks old) were obtained. All animal maintenance and procedures were carried in accordance with the National Institute of Health Guide for the Care and Use of Laboratory Animals, with the approval of the Animal Research Committee of Dalian Medical University. A375 melanoma cells (2 × 10^6^) were inoculated subcutaneously into the flank of the nude mice. Mice were randomly divided into 4 groups (5 mice per group): DMSO, melatonin (25 mg/kg), vemurafenib (20 mg/kg) and melatonin + vemurafenib, which were started 5 days after injection and co-treatment every other day for 2 weeks. The tumor volume in mm^3^ was calculated as V = (width^2^ × length)/2 using digital calipers and the tumor weight was recorded after the mice were sacrificed. Partial tumor was lysed for protein expression analysis through western blot analysis, and partial was sliced and fixed in formalin and embedded in paraffin for protein expression analysis through the immunohistochemical staining.

### Immunohistochemistry staining

Briefly, Tumors were dissected and fixed in 10% formalin overnight, embedded in paraffin, and incised to 4 um thick. Immunohistochemistry (IHC) staining was performed using following the DAB Kit (Origene, China). The primary antibodies iNOS, hTERT, p-p65, Epcam, CD44, PCDNA were used with indicated dilution ratio. Sections were stained with hematoxylin to recognize nucleus.

### Statistical analysis

Data are represented as mean ± standard deviation (SD). Analysis of variance and Student’s t test were used to compare the values of the test and control samples. *P* < 0.05 was considered to be a statistically significant difference. SPSS17.0 software was used for statistical analysis, and all the experiments were done three times.

## Results

### Melatonin potentiated vemurafenib-mediated inhibition of cell proliferation via cell cycle arresting

**Fig. 1:**
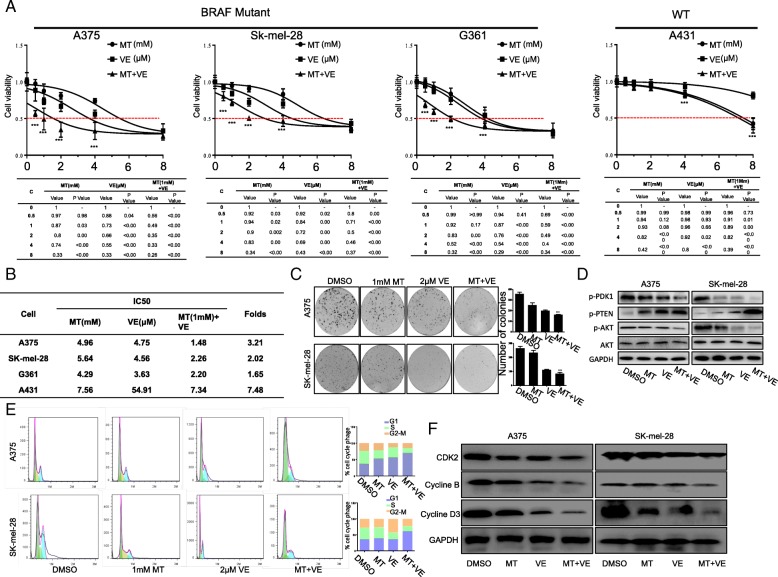
Melatonin enhanced the inhibition of cell proliferation by vemurafenib. (**a**). Human melanoma cells were treated with the increasing doses of vemurafenib (VE), melatonin (MT) alone or combined for 48 h, and the cell viability was examined by MTT assay. (**b**). The IC_50_ values of vemurafenib (VE) for cell viability inhibition in cells treated with or without melatonin (MT) were determined. (**c**). Clone formation in A375 cells and SK-mel-28 cells treated with vemurafenib (VE) (2.5 μM) and melatonin (MT) (1.0 mM) for 48 h were observed and the colony number were quantified. (**d**). AKT signaling-associated protein markers: PDK1, p-PTEN, p-AKT and AKT was respectively detected by Western blot assay in melanoma cells with indicated treatment. (**e**). DNA content-based cell cycle analysis was carried out in melanoma cells treated with VE or MT alone or their combination for 48 h. The percentage of cells at each phase of cell cycle was quantified. (**f**). G1/S checkpoint pathway was detected by Western blot assay in melanoma cells with indicated treatment. The data is presented as mean ± SD of three separate experiments, ^*^P < 0.05, ^**^P < 0.01, significant differences compared to the control groups

To assess the effect of melatonin on vemurafenib-mediated inhibition of cell proliferation, we tested cell viability in a panel of human melanoma cell lines. The expression of BRAF V600E mutant protein was firstly determined in different cell lines (Additional file 1: Figure S1A). Cells were treated with vemurafenib (0, 0.5, 1, 2, 4, or 8 μM) or various concentrations of melatonin (0, 0.5, 1, 2, 4, or 8 mM) for 24 h. Vemurafenib significantly decreased the cell viability compared with the untreated control in melanoma cell lines with BRAF V600E mutant, and combined treatment with melatonin significantly enhanced the suppression of cell viability in a dose-dependent manner compared with the treatment with vemurafenib alone (Fig. 1a). By contrast, no improved suppression was observed in A431 cells with wild-type BRAF when they were co-treated by vemurafenib and melatonin (1 mM) by comparison with the treatment with vemurafenib alone (Fig. 1a). We next determined the half-maximum inhibitory concentration (IC_50_) together with cell viability assays (Fig. 1b), and found that melanoma cells with BRAF mutant (V600E) under co-treatment of vemurafenib and melatonin was more sensitive to vemurafenib. We next established A375 cell line with the relative vemurafenib resistance, A375R (Additional file 1: Figure S1B), and similarly found that the combined use of vemurafenib and melatonin significantly decreased IC_50_ of vemurafenib (Additional file 1: Figure S1C, D). Furthermore, the combination of vemurafenib and melatonin also exhibited lower colony formation ratio by comparison with the cells treated with the single agent (Fig. 1c). In addition, we assessed the effects of drug treatment on AKT signaling, which may be involved in cell proliferation inhibition. We found the combinational drug treatment caused a significant decrease of p-PDK1 and p-AKT and increase in levels of p-PTEN (Fig. 1D). These findings suggested that melatonin sensitized vemurafenib-mediated anti-proliferative function by targeting AKT signaling.

Cell cycle is closely related with tumor intensive proliferation and subdued apoptosis. To assess whether cell cycle was implicated in the synergistic activity of vemurafenib and melatonin on cell viability, FACS analysis was performed. Treatment with melatonin (1 mM) alone increased the number of cells at G1-phase while vemurafenib (2.5 μM) alone significantly increased the sub-G1 cell population. Importantly, combination of melatonin (1 mM) with vemurafenib (2.5 μM) significantly enhanced the induction of cell cycle arrest at G1-phase compared with the treatment with vemurafenib alone (Fig. 1e). Consistently, combined treatment of vemurafenib (2.5 μM) and melatonin (1 mM) caused a decrease of cyclin B, cyclin D3 and CDK2 (Fig. 1f).

### Melatonin enhanced vemurafenib-mediated inhibition of cell migration and invasion in melanoma cells

**Fig. 2:**
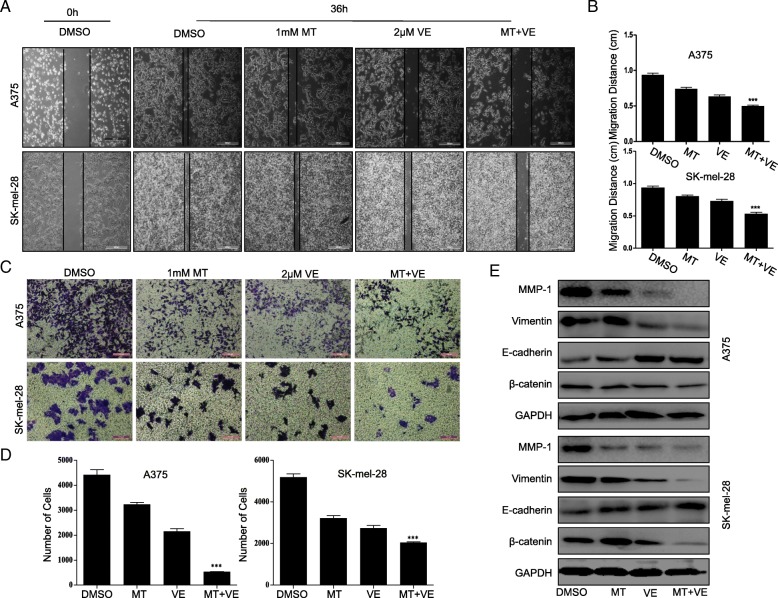
Melatonin enhanced the inhibition of cell migration and invasion by vemurafenib’ (**a**). Cell migration was analyzed by a scratch assay. A375 cells and SK-mel-28 cells were grown to full confluency. The cell monolayers were wounded with a sterile pipette tip, and washed with medium to remove detached cells from the plates. Then the cells were treated with vemurafenib (VE) (2.5 μM), melatonin (MT) (1.0 mM) or combinational treatment. After 36 h, the wound gap was observed and photographed. (**b**). The distance of migration cells were calculated relative to the original gap. (**c**). Cell invasion was analyzed by a transwell assay, and the invaded cells were stained and observed. (**d**). The invasion cells number were presented. (**e**). EMT markers: MMP1, Vimentin, E-cadherin and β-caternin was respectively detected by Western blot assay in melanoma cells with indicated treatment.*P < 0.05, significant differences between the VE + MT-treated groups and the VE-treated groups

We also evaluated the influence of the combined treatment with melatonin and vemurafenib on the cell migration and invasion ability in melanoma cells. The scratch assay was employed to determine the combined effect of vemurafenib with melatonin on cell migration in melanoma cells. We found that the part of gap or wounding space between cell layers was occupied almost or completely by the migrating cells after 36 h in the control group and the group with melatonin treatment alone (Fig. 2a). The treatment with vemurafenib (2.5 μM) alone inhibited cell migration. However, the combined treatment with melatonin (1.0 mM) markedly enhanced vemurafenib-mediated inhibition of cell migration in melanoma cells (Fig. 2a, b). These results suggested that the combinational use of vemurafenib and melatonin showed more potential effect in suppressing cell migration in melanoma cells. Consistent with cell migration inhibition, the combinational use of vemurafenib and melatonin led to a statistically significant decrease in melanoma cells in metastasis and invasiveness compared with the single compound treatment (Fig. 2c, d). In addition, the combination caused a decrease of Vimentin and β-caternin and increase in levels of E-cadherin (Fig. 2e). In agreement with this, the elevated migration and invasion inhibition upon co-treatment with vemurafenib and melatonin compared to vemurafenib treatment alone was identified in A375R cells (Additional file 1: Figure S2A, B). These findings suggested that the combination of vemurafenib and melatonin might suppress epithelial-mesenchymal transition (EMT) of melanoma cells.

### Melatonin increased vemurafenib-induced apoptosis by modulating cytochrome c and caspase signaling pathway

**Fig. 3:**
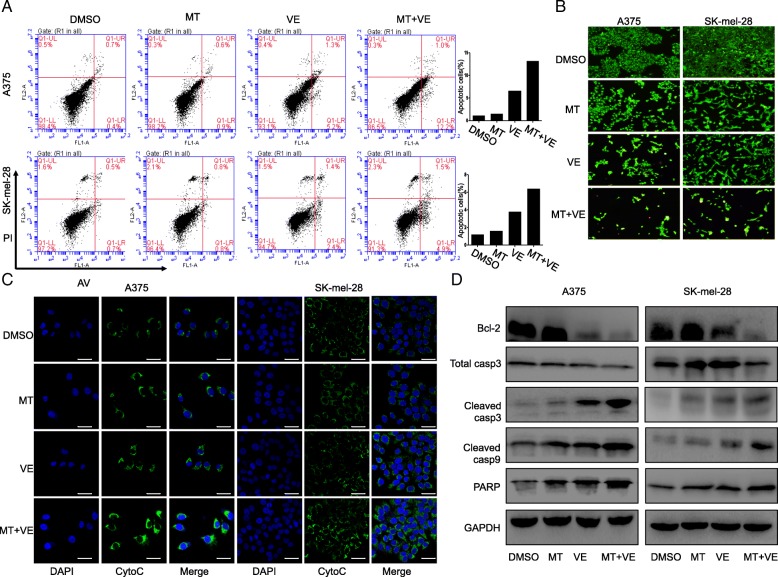
Melatonin increased apoptosis induced by vemurafenib via the cytochrome c/caspase signaling pathway. Human melanoma cells were treated with vemurafenib (VE) (2.5 μM) and melatonin (MT) (1.0 mM) for 24 h. (**a**). The apoptosis was then determined by a FACS analysis. (**b**). Acridine orange/ethidium bromide fluorescence staining was performed in melanoma cells. (**c**). The release of cytochrome c (cyto-c) was monitored by immunofluorescence imaging analysis from the inter-mitochondrial space into the cytosol. (**d**). The levels of the Bcl-2, cleaved, caspase-3, 9 and PARP proteins were analyzed by Western blotting. The apoptosis are represented by relative percentages of apoptotic cells versus that in DMSO-treated cells

To assess whether the synergistic inhibition of vemurafenib and melatonin on cell growth in melanoma cells is associated with cell apoptosis, we confirmed the pro-apoptotic function of such combinational use by FACS analysis. As shown in Fig. 3a, treatment with vemurafenib alone at the doses of 2.5 μM induced 6.5 and 3.8% apoptotic cells in A375 and SK-mel-28 cells respectively. However, the addition of melatonin (1 mM) greatly increased vemurafenib-induced apoptosis, resulting in a 13.2 and 6.4% induction of apoptosis in melanoma cells respectively at 24 h after treatment (Fig. 3a).

We further compared the damage extent of cells co-treated with vemurafenib and melatonin by AO/EB staining. The phenomenon that more EB passed through damaged cell membrane, embedded in nucleus DNA, and much brighter orange red fluorescence accumulated in the nucleus was observed in the group treated with combined compounds (Fig. 3b), indicating that more apoptosis was induced in melanoma cells when they were exposed to vemurafenib and melatonin simultaneously but not to single compound alone.

Moreover, we performed immunofluorescence (IF) analysis to monitor the subcellular localization of cytochrome c, which is an upstream molecule of the caspase cascade-dependent apoptotic signaling pathway. We found that treatment with vemurafenib (2.5 μM) or melatonin (1 mM) alone for 24 h triggered the release of cytochrome c from the inter-mitochondrial space into the cytosol in melanoma cells, but the combined treatment with these two drugs markedly elevated the release of cytochrome c (Fig. 3c). Importantly, we also observed that melatonin (1 mM) remarkably promoted vemurafenib-induced activation of cleaved caspase-3, cleaved caspase-9 and inactivation of Bcl-2 as well as cleavage of PARP in melanoma cells (Fig. 3d). Collectively, melatonin increased vemurafenib-induced pro-apoptosis by modulating cytochrome c and caspase signaling pathway in melanoma cells.

### Melatonin enhanced vemurafenib-induced inhibition of iNOS expression by inhibiting NF-κB signaling pathway

**Fig. 4:**
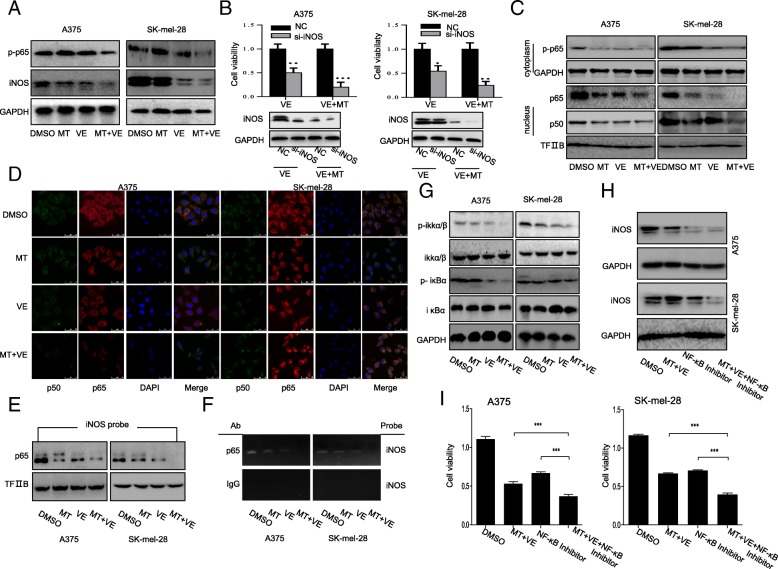
Melatonin enhanced the vemurafenib-induced inhibition of INOS expression by inhibiting NF-κB signaling pathway. (**a**). The expression level of iNOS and p-P65 protein were analyzed by Western blot in human melanoma cells treated with the indicated doses of vemurafenib (VE) (2.5 μM) and melatonin (MT) (1.0 mM) for 48 h. (**b**). Cell viability was analyzed by MTT assay in melanoma cells treated with vemurafenib (2.5 μM) combined with or without MT (1.0 mM) for 24 h after pretreatment with the iNOS targeting siRNA for 48 h. (**c**). The expression of p50/p65 was determined from nucleus extracts prepared from melanoma cells by Western blotting. (**d**). The subcellular localization of p50 and p65 and their co-localization in human melanoma cells treated with 2.5 μM vemurafenib (VE) and 1.0 mM melatonin (MT) for 48 h were examined by confocal microscopy. Cells with typical morphology were presented from more than 100 cells at each experiment. (**e**). The streptavidin-biotin pulldown assay was performed to analyze the binding of P65 protein to iNOS promoter in melanoma cells with the indicated treatment. (**f**). Binding of p65 to the iNOS promoter in chromatin structure by ChIP assay. IgG, a negative control for ChIP in melanoma cells with the indicated treatment. (**g**). Human melanoma cells were treated with 2.5 μM vemurafenib (VE) and 1.0 mM melatonin (MT). At 48 h after treatment, the IKKβ, p-IKKβ, IκBα and p-IκBα proteins were analyzed by Western blotting. (**h**). Vemurafenib (VE) combined with or without 1.0 mM melatonin (MT) followed by NF-κB inhibitor treatment and then iNOS expression and cell viability was respectively analyzed Western blot. (**i**). MTT assay in melanoma cells treated with NF-κB Activation Inhibitor followed by the treatment of vemurafenib (VE) combined with or without 1.0 mM melatonin (MT). Each data point was calculated from three triplicate groups and the data is presented as the mean ± SD. ^*^P < 0.05, significant difference between treatment group and control group

Inducible nitric oxide synthase (iNOS), one of pro-inflammatory cytokines, which is also the direct downstream effectors of NF-κB, has been previously identified as important inducers of cell proliferation, migration, and angiogenesis in melanoma cells. In order to identify the involvement of iNOS expression and the further mechanistic basis in melatonin-sensitized proliferative inhibition mediated by vemurafenib, we analyzed the effect of combinational treatment on iNOS expression. We found that vemurafenib (2.5 μM) alone decreased the expression of iNOS. However, co-treatment with vemurafenib (2.5 μM) and melatonin (1 mM) almost diminished the expression of iNOS compared with vemurafenib treatment itself (Fig. 4a). In addition, treatment with iNOS si-RNA alone showed inhibitory effect on cell viability, however, the combined treatment with melatonin, vemurafenib and si-iNOS markedly increased such inhibition (Fig. 4b). These results suggested that the elevated proliferative inhibition by co-treatment with melatonin and vemurafenib might partially be realized through inhibiting the activation of iNOS signaling.

It has also been shown that NF-κB activation protects cells from BRAF inhibition-induced cell death. We next determined the level of p50/p65 in the cell lysate, and found that vemurafenib (2.5 μM) or melatonin (1 mM) treatment alone reduced the expression of p50/p65 in the nuclei of melanoma cells (Fig. 4c), and the treatment with melatonin and vemurafenib markedly inhibited translocation of the NF-κB p65/p50 proteins from cell cytoplasm to nucleus by comparison with the control treatment group (Fig. 4c, d). Furthermore, we determined the effect of the combinational treatment on the binding of p65 at iNOS promoter region. Pull down and ChIP assay indicated that the binding of p65 to the iNOS promoter was almost diminished after co-treatment with melatonin and vemurafenib (2.5 μM) compared to the single compound treatment alone (Fig. 4e, f). These results indicate that the inhibition of iNOS expression in melanoma cells by combinational use of melatonin and vemurafenib might be mediated by inhibiting the translocation of NF-κB p50/p65 from cytoplasm to cell nuclei and further inhibiting their binding at iNOS promoter.

Moreover, to better understand the involvement of NF-κB signaling pathway in the iNOS expression inhibition mediated by the co-treatment of melatonin and vemurafenib in melanoma cells, we then investigated the upstream signaling molecules of NF-κB pathway. As shown in Fig. 4g, the combinational treatment not only significantly suppressed the phosphorylation of IKKβ in melanoma cells without affecting its overall expression, but also decreased the expression level of phosphorylated IκBα. Next, we investigated the effect of NF-κB inhibitor on iNOS expression and cell viability upon co-treatment with melatonin and vemurafenib. The results indicated compared to single melatonin and vemurafenib co-treatment, NF-κB signaling inhibition caused more iNOS expression suppression and cell viability inhibition (Fig. 4h, i). Collectively, NF-κB signaling pathway was a potential target of co-treatment with melatonin and vemurafenib in melanoma cells to finally suppress iNOS expression.

### Melatonin enhanced vemurafenib-induced inhibition of cancer stem cell traits by down-regulating hTERT in melanoma cells

**Fig. 5:**
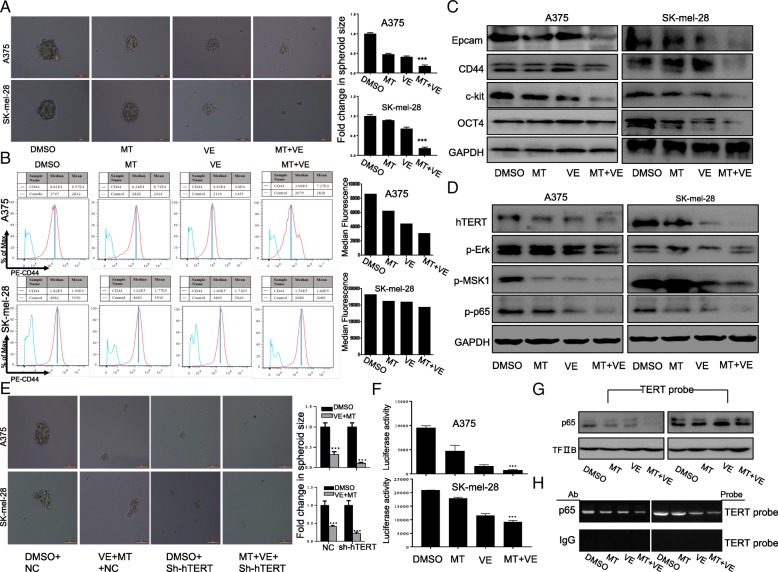
Combination of vemurafenib and melatonin inhibited cancer stem cell traits by down-regulating hTERT in melanoma cells. Human melanoma cells were exposed to vemurafenib (VE) (2.5 μM) with or without melatonin (MT) (1.0 mM) for 48 h. (**a**). The representative images of tumor sphere formation of melanoma cells with indicated treatment. (**b**). CD44 expression on the surface of melanoma cells was analyzed by FACS. (**c**). The expression of CSC-related markers Epcam, CD44, c-kit and Oct4 were determined by western blot in A375 and SK-mel-28 cells with the indicated treatment. (**d**). The expression of hTERT-p-MSK1-p65 pathway were determined by western blot in A375 and SK-mel-28 cells with the indicated treatment. (**e**). The representative images of tumor sphere formation of melanoma cells treated with DMSO or vemurafenib (2.5 μM) combined with MT (1.0 mM) for 24 h after pretreatment with the hTERT targeting shRNA. (**f**). Melanoma cells were co-treated with the plasmids of hTERT promoter driven-luciferase and vemurafenib (VE) (2.5 μM) with or without melatonin (MT) for 48 h followed by a dual-luciferase assay. The relative luciferase intensity per mg protein was calculated in the treated cells. (**g**). The streptavidin-biotin pulldown assay was performed to analyze the binding of P65 protein to hTERT promoter in melanoma cells with the indicated treatment. (**h**). Binding of p65 to the hTERT promoter in chromatin structure by ChIP assay. IgG, a negative control for ChIP in melanoma cells with the indicated treatment. The data are presented as the mean ± SD of three separate experiments. ^*^P < 0.05, significant differences between treatment groups and DMSO control groups

Next, to assess the effect of the combinational treatment on the stemness of melanoma cells, tumor-sphere model was performed and we found melatonin significantly increased the inhibition of vemurafenib on the tumorsphere size in both A375 and SK-mel-28 cells (Fig. 5a). Consistently, cell-surface phenotype analysis indicated that the combinational treatment decreased CD44 expression on the surface of melanoma cells (Fig. 5b). To further investigate the mechanism of the combinational effect related to melanoma stem cell traits, we determined the expression levels of cancer stem cell (CSC)-related markers, including Epcam, CD44, OCT4 and c-kit. The co-treatment of melatonin and vemurafenib remarkably inhibited their expression (Fig. 5c).

Given the critical role of MAPK pathway in BRAF-targeting therapy resistance and the existence of Erk-MSK1-P65 signaling as the alternative pathway of MAPK pathways, we then evaluated the effect of the co-treatment on this pathway and found vemurafenib combined with melatonin remarkably down-regulated their phosphorylated level by comparison to vemurafenib or melatonin treatment alone (Fig. 5d). Moreover, the expression of hTERT, which is mutually stabilized by p65 acting as a molecular buttress in catalytically active conformations, and plays an important role in the maintenance of stem cell traits, was also significantly inhibited by the combinational treatment (Fig. 5d). Treatment with hTERT sh-RNA alone similarly showed inhibitory effect on the tumorsphere number and size, however, the combined treatment with melatonin, vemurafenib and hTERT sh-RNA markedly increase such inhibition (Fig. 5e), further confirming the involvement of hTERT in the stemness weakening mediated by the combined use of melatonin and vemurafenib.

We then assessed the effect of the combinational treatment on hTERT promoter activity in melanoma cells, and found that co-treatment of melatonin and vemurafenib markedly attenuated the expression of hTERT promoter-driven luciferase as compared with control group or single drug treatment group (Fig. 5f). To further provide mechanistic insights into hTERT expression regulated by the combinational treatment of melatonin and vemurafenib, we explored the possibility of p65 participating in the hTERT expression in melanoma cells treated by melatonin and vemurafenib. Pull down (Fig. 5g) and ChIP assay (Fig. 5h) indicated that the binding of p65 to the hTERT promoter was almost diminished after co-treatment with melatonin and vemurafenib compared to the single compound treatment alone. These results indicate that the inhibition of hTERT expression in melanoma cells by combinational use of melatonin and vemurafenib might be mediated by inhibiting the binding of p65 at hTERT promoter.

### Inhibition of tumor growth and CSC properties by melatonin and vemurafenib in mouse model with melanoma xenografts

**Fig. 6:**
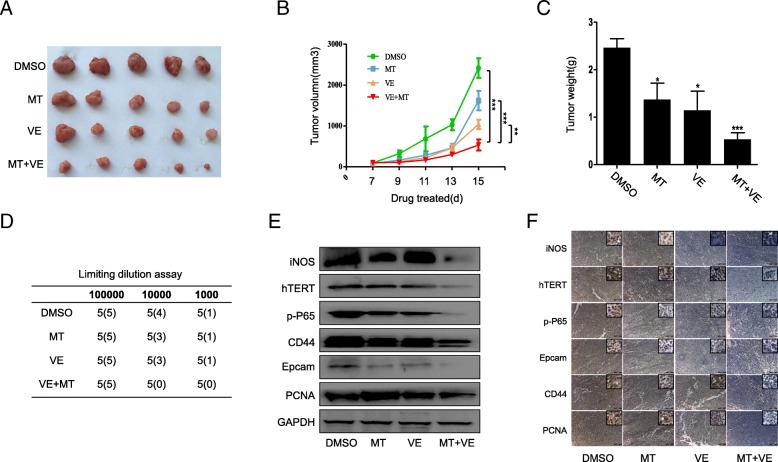
Combination of vemurafenib and melatonin inhibited tumor growth and CSCs properties in mice. (**a**). The morphology of tumor xenografts of each nude mice after anatomy at 15 days of treatment. (**b**). Tumor volume of each group of nude mice was measured and calculated as V = (width2 × length)/2. (**c**). Tumor weight of each group of nude mice was measured, ^*^P < 0.05. (D). Limiting dilution assays was performed and the finally formed tumors were calculated. (**e**-**f**). The expression levels o of iNOS, hTERT, p65, CD44, Epcam, PCNA expression from tumor exnografts in each group of nude mice by western blot (**e**) and immunohistochemistry assay (**f**). The data in panels are presented as the mean ± SD of three tests. *P* < 0.05, significant differences between treatment groups and DMSO control groups *n* = 5 mice/group. Magnification, 100 ×

We also validated the combinational treatment-mediated regulation of tumor growth in a mouse model with melanoma xenografts. A375 cells were injected into nude mice (flank), and we found the mice with subcutaneous xenografts of melanoma cells displayed remarkable tumor growth delay when they were co-treated with melatonin and vemurafenib (Fig. 6a, b, c). Limiting dilution assays showed no tumor was formed in combined treatment group when cell number injected was 1 × 10^4^ (Fig. 6d). Further, Western blot and immunohistochemistry analyses showed that combination of melatonin and vemurafenib not only remarkably suppressed the expression of hTERT, iNOS, p65, CD44 and Epcam, but also decreased the level of PCNA in xenografts, compared with the single drug treatment or control group (Fig. 6e, f). These results demonstrated again the improved antitumor effect of vemurafenib upon co-use with melatonin in melanoma treatment, and also confirmed the involvement of p65/iNOS/hTERT signaling in the co-treatment-mediated regulation of melanoma growth and CSC expansion.

## Discussion

### Synergistic antitumor effect of melatonin and vemurafenib

Chapman PB et al. [36] reported the response initially to vemurafenib treatment by approximately 80% of patients with mutant BRAF melanomas, but acquired drug resistance develops in the majority of patients commonly within 1 year. In this study, we demonstrated that melatonin sensitized vemurafenib-mediated antitumor effect in melanoma cells, which was represented by the improved cell viability suppression, clonogenicity, migration and invasion inhibition, apoptosis induction and stemness attenuation. The IC_50_ value of vemurafenib was dramatically decreased under co-treatment with melatonin in comparison with vemurafenib treatment alone. The increased G1 phase among melanoma cells demonstrated cell cycle arrest might contribute to the enhanced proliferation inhibition caused by co-treatment with vemurafenib and melatonin. In addition, we also demonstrated the up-regulated expression inhibition of iNOS and hTERT upon co-treatment of melatonin and vemurafenib in melanoma cells, clarifying the possible molecular mechanisms of such combinational treatment in enhancing anti-cancer effects.

### Synergistic inhibition of iNOS signaling by melatonin and vemurafenib

Inducible NO synthases (iNOS), combined with its product NO, has shown high expression level in melanoma, and has been reported to be a strong predictor about disease-specific and overall survival (OS) in stage III melanoma patients [37]. The iNOS signaling was also identified in the regulation of proliferation, migration invasion and apoptosis in many kinds of cancer cells. Therefore, the effective therapeutic strategy targeting iNOS has been developed and is expected to provide therapeutic implications in cancer treatment. Our study found the enhanced inhibition of iNOS expression in melanoma cells after co-treatment with vemurafenib and melatonin in comparison with single agent treatment. Moreover, iNOS knockdown caused more significant proliferative inhibition in melanoma cells mediated by the two compounds co-treatment, suggesting that inhibition of iNOS signaling at least partially contributed to the sensitization potential of melatonin in vemurafenib-mediated cell proliferation inhibition in melanoma cells.

### Synergistic inhibition of NF-κB signaling by melatonin and vemurafenib

It has been reported that NF-κB activation protects cells from BRAF inhibition-induced cell death. Similarly, the reactivation of the PI3K/Akt-CREB-AEBP1-NF-κB pathway showed to contribute to BRAF inhibitor-resistant phenotype in melanoma treatment [38, 39]. Given that iNOS expression is classically regulated by NF-κB signaling, we therefore initially focused on its participation in venurafenib-mediated regulation of iNOS expression. Our study identified co-treatment with melatonin and venurafenib inhibited the NF-κB signaling by dephosphorylating Ikkα (IκB kinase). Furthermore, vemurafenib combined with melatonin dephosphorylated the NF-κB subunit p65, decreased its translocation from cytoplasm into nucleus, abrogated the binding of the NF-κB complex to the iNOS promoter and finally led to its expression suppression. Collectively, our results clearly show NF-κB signaling behaves as a dependent middle bridge in the increased iNOS expression regulation mediated by the combinational use of melatonin and vemurafenib in melanoma treatment.

### Synergistic inhibition of hTERT signaling by melatonin and vemurafenib

Accumulating evidence demonstrated the significant role of cancer stem cells in cancer development [40, 41]. hTERT was found to be involved in the maintenance of stemness in cancer cells [42, 43]. The relationship between high expression of hTERT and inferior outcome in patients with melanoma was reported recently [44]. Combined with the fact that melatonin plays its antitumor effect by affecting hTERT expression and telomerase activity [45, 46]. Hence, we deduce the improved antitumor effects displayed by the combinational treatment of melatonin and vemurafenib might be partially realized by the enhanced stemness abrogation through targeting at hTERT expression. Our study showed that the combination of melatonin and vemurafenib decreased the level of stemness markers, including CD44, c-kit and OCT4, and attenuated the expression of hTERT by impairing induction of hTERT promoter activity mediated by p65. Notably, hTERT silencing led to more significant stemness arrest in melanoma cells mediated by co-treatment with melatonin and vemurafenib, implying the potentiating effect of melatonin on vemurafenib-induced stemness inhibition through the p65/hTERT signaling pathways, and such signaling inhibition may be a striking target for potential therapeutics in melanoma.

Given that iNOS was similarly reported to contribute to the promotion of cancer stem cell phenotype [47, 48], and the combinational treatment of melatonin and vemurafenib induced more expression inhibition of iNOS compared to single drug treatment, it is reasonable that besides hTERT, iNOS might also be involved in the abolished stemness maintenance caused by the combined drug application. In agreement with this, besides iNOS, most likely, hTERT also mediates the improved proliferation suppression caused by the combined drug treatment. All these possibilities deserve better explorations in our further study.

## Conclusion

**Fig. 7:**
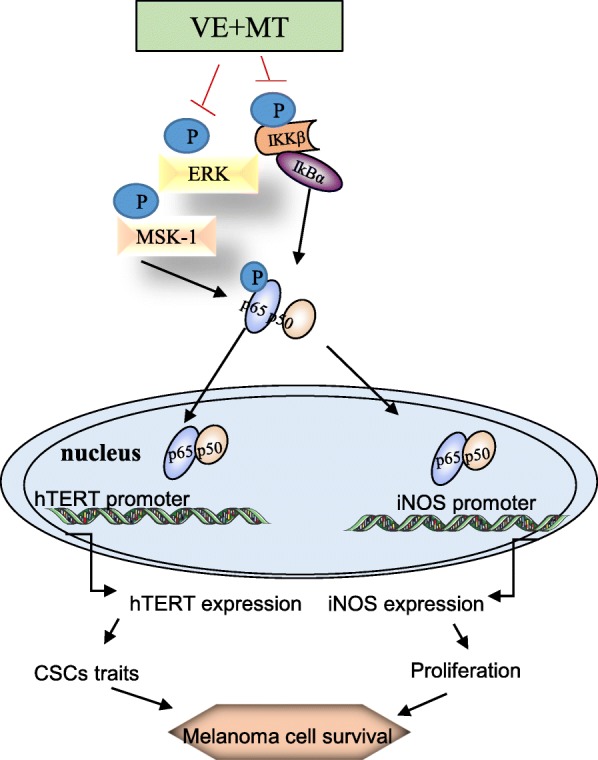
The schematic diagram of the molecular mechanisms by which melatonin synergizes vemurafenib in melanoma treatment. The symbol (⊦) indicates negative regulation. The arrow (→) indicates direct or indirect positive regulation

In summary, our findings demonstrated that melatonin potentiated the vemurafenib-mediated antitumor effect in melanoma. The combination led to melanoma cell growth inhibition in vitro and in vivo, as evidenced by decreased proliferative and invasive capacity, and enhanced cell cycle arrest and apoptosis induction. Moreover, we provided mechanistic insight into the combination, and found that melatonin synergizes the antitumor effect of vemurafenib by inhibiting the iNOS/hTERT signaling and cancer-stem cell traits in human melanoma (Fig. 7). Our findings therefore demonstrated the potential of melatonin, not only in antagonizing the toxicity of vemurafenib but also in augmenting its sensitivities in melanoma treatment. Therefore, the combinational use of a natural endogenous hormone (melatonin) and a small molecule inhibitor (vemurafenib) targeting BRAF should be considered as a promising therapeutic strategy to break drug resistance in melanoma treatment.

## Additional file

Additional file 1: **Figure S1.** Melatonin enhanced the inhibition of cell proliferation by vemurafenib. (A). BRAF V600E, p-ERK and ERK was respectively detected by Western blot assay in melanoma cells (A375, SK-mel-28, G361 and A431). (B). ABCG2 was respectively detected by Western blot assay in A375 and A375R cells**. (C).** Human melanoma cells (A375R) were treated with the increasing doses of vemurafenib (VE), melatonin (MT) alone or their combination for 48 h, and the cell viability was examined by MTT assay. (D). The IC_50_ values of vemurafenib (VE) for cell viability inhibition in A375R cells treated with or without melatonin (MT) were determined. **Figure S2.** Melatonin enhanced the inhibition of cell migration and invasion by vemurafenib (A). Cell migration was analyzed by a scratch assay. A375R cells were treated with vemurafenib (VE) (4 μM), melatonin (MT) (1.0 mM) or their combination. After 36 h, the wound gap was observed and photographed, and the distance of migration cells were calculated relative to the original gap. (B). Cell invasion was analyzed by a transwell assay in A375R cells with different treatment. The invaded cells were stained and observed, and the number of the invasion cells was presented. The data is presented as mean ± SD of three separate experiments, ^*^P < 0.05, ^**^P < 0.01, significant differences compared to the control groups. (PDF 4326 kb)

## Abbreviations

BRAF: a protein called B-raf
FBS: Fetal bovine serum
hTERT: human telomerase reverse transcriptase
IHC: Immunohistochemistry
iNOS: Inducible NO synthases
MAPK: mitogen-activated protein kinase
