# Correction: Melatonin synergizes BRAF-targeting agent vemurafenib in melanoma treatment by inhibiting iNOS/hTERT signaling and cancer-stem cell traits

**DOI:** 10.1186/s13046-024-03104-w

**Published:** 2024-06-26

**Authors:** Jiaojiao Hao, Wenhua Fan, Yizhuo Li, Ranran Tang, Chunfang Tian, Qian Yang, Tianhua Zhu, Chaoliang Diao, Sheng Hu, Manyu Chen, Ping Guo, Qian Long, Changlin Zhang, Ge Qin, Wendan Yu, Miao Chen, Liren Li, Lijun Qin, Jingshu Wang, Xiuping Zhang, Yandong Ren, Penghui Zhou, Lijuan Zou, Kui Jiang, Wei Guo, Wuguo Deng

**Affiliations:** 1 Institute of Cancer Stem Cells and The Second Affiliated Hospital, Dalian Medical University, Dalian, China; 2 State Key Laboratory of Oncology in South China; Collaborative Innovation Center of Cancer Medicine, Sun Yat-Sen University Cancer Centre, Guangzhou, China; 3 Nanjing Maternity and Child Health Care Hospital, Women’s Hospital of Nanjing Medical University, Nanjing, China; 4 Sun Yat-Sen Memorial Hospital of Sun Yat-Sen University, Guangzhou, China; 5 Cloud Health Genomics Ltd, Shanghai, China

**Correction** **:** **J Exp Clin Cancer Res 38, 48 (2019)**

**https://doi.org/10.1186/s13046-019-1036-z**

Following publication of the original article [1], authors identified errors in Figure 3c and Figure 5e. The merged image of VE group in sk-mel-28 cells (Fig. 3C) and the image of DMSO + NC group in A375 cells (Fig. 5E) has been mistakenly uploaded, respectively.

**Incorrect Figure 3**

**Fig. 3:**
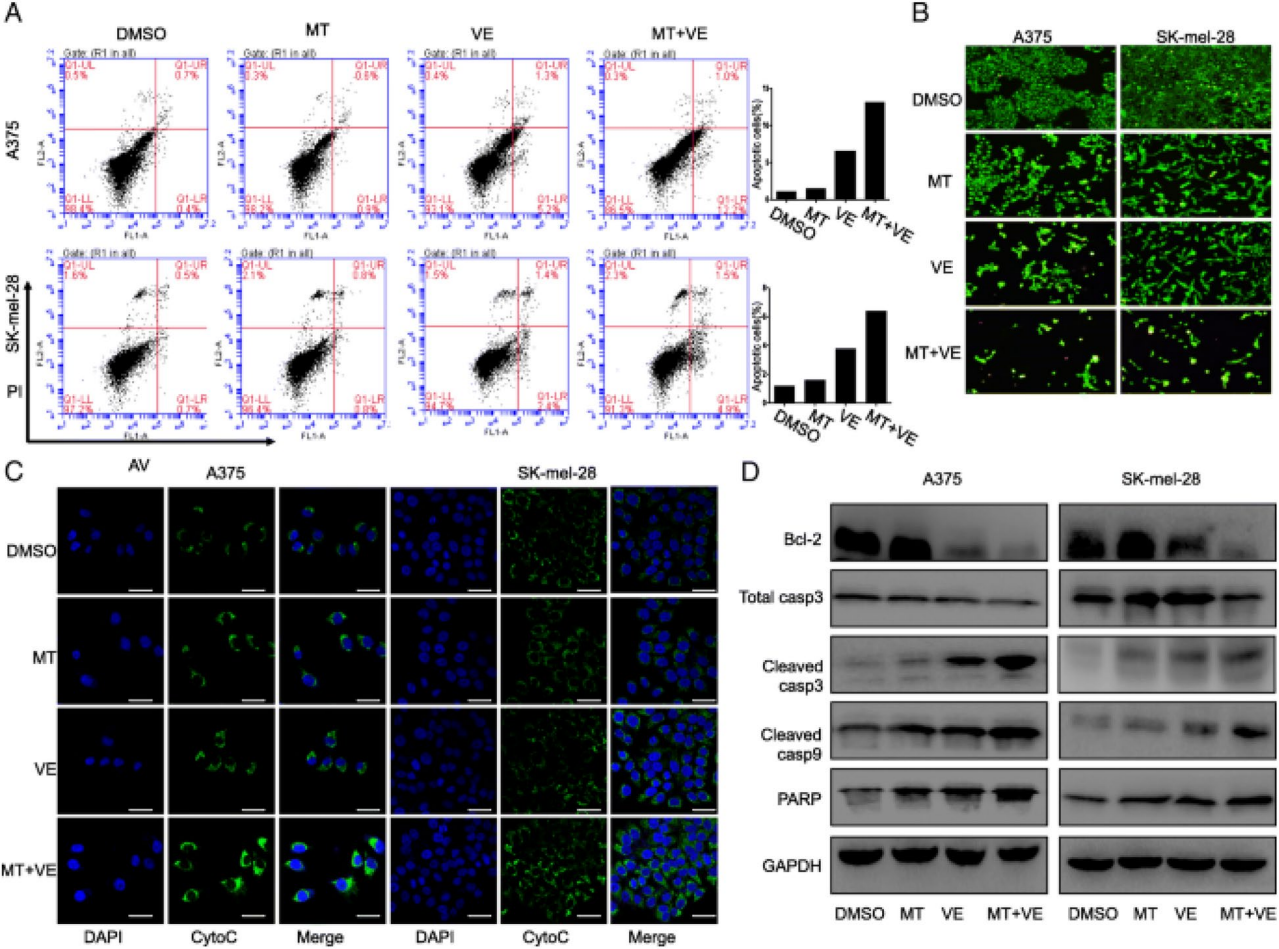
Melatonin increased apoptosis induced by vemurafenib via the cytochrome c/caspase signaling pathway. Human melanoma cells were treated with vemurafenib (VE) (2.5 μM) and melatonin (MT) (1.0 mM) for 24 h. (**a**). The apoptosis was then determined by a FACS analysis. (**b**). Acridine orange/ethidium bromide fluorescence staining was performed in melanoma cells. (**c**). The release of cytochrome c (cyto-c) was monitored by immunofluorescence imaging analysis from the inter-mitochondrial space into the cytosol. (**d**). The levels of the Bcl-2, cleaved, caspase-3, 9 and PARP proteins were analyzed by Western blotting. The apoptosis are represented by relative percentages of apoptotic cells versus that in DMSO-treated cells

**Correct Figure 3**

**Fig. 3:**
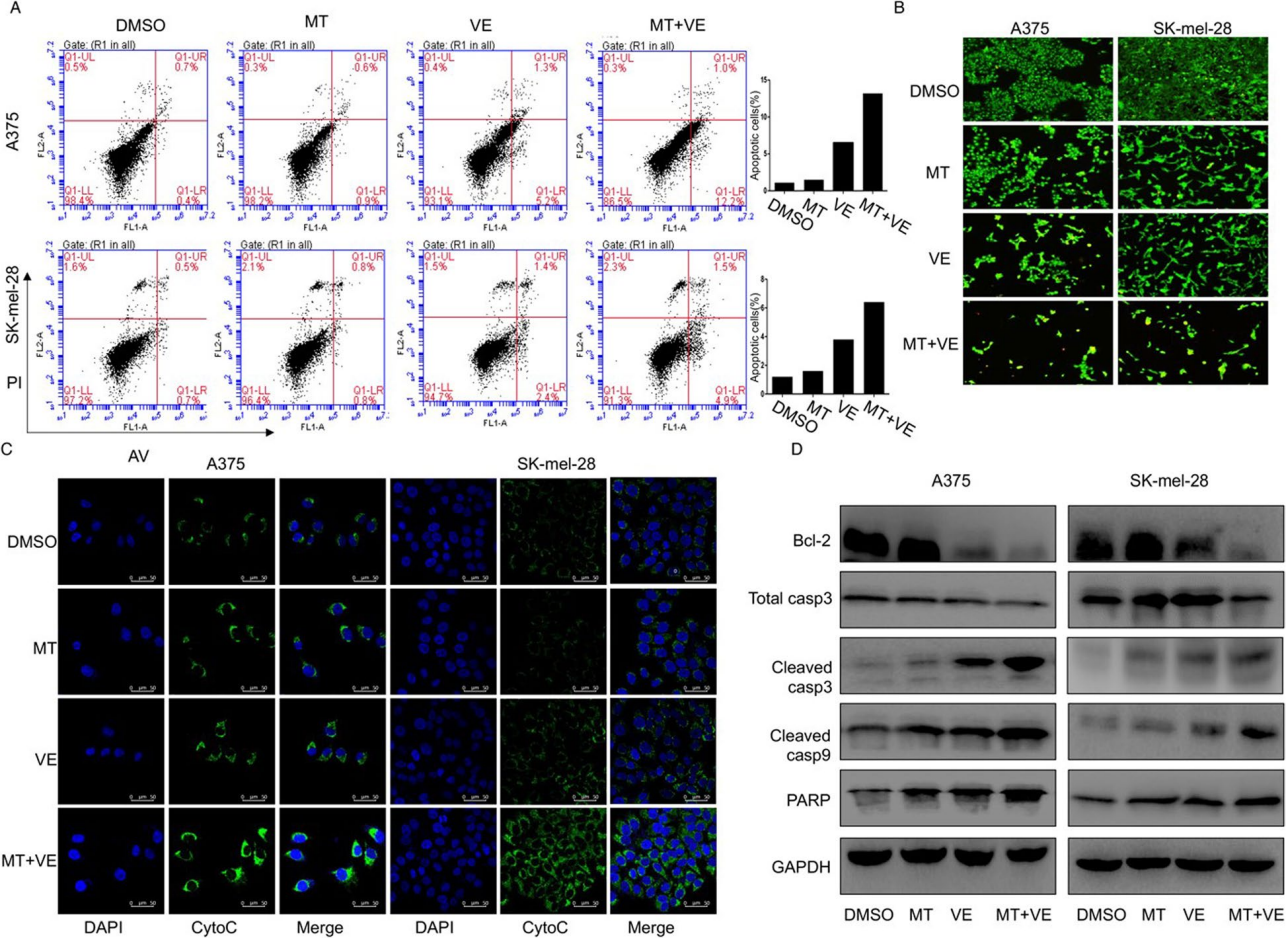
Melatonin increased apoptosis induced by vemurafenib via the cytochrome c/caspase signaling pathway. Human melanoma cells were treated with vemurafenib (VE) (2.5 μM) and melatonin (MT) (1.0 mM) for 24 h. (**a**). The apoptosis was then determined by a FACS analysis. (**b**). Acridine orange/ethidium bromide fluorescence staining was performed in melanoma cells. (**c**). The release of cytochrome c (cyto-c) was monitored by immunofluorescence imaging analysis from the inter-mitochondrial space into the cytosol. (**d**). The levels of the Bcl-2, cleaved, caspase-3, 9 and PARP proteins were analyzed by Western blotting. The apoptosis are represented by relative percentages of apoptotic cells versus that in DMSO-treated cells

**Incorrect Figure 5**

**Fig. 5:**
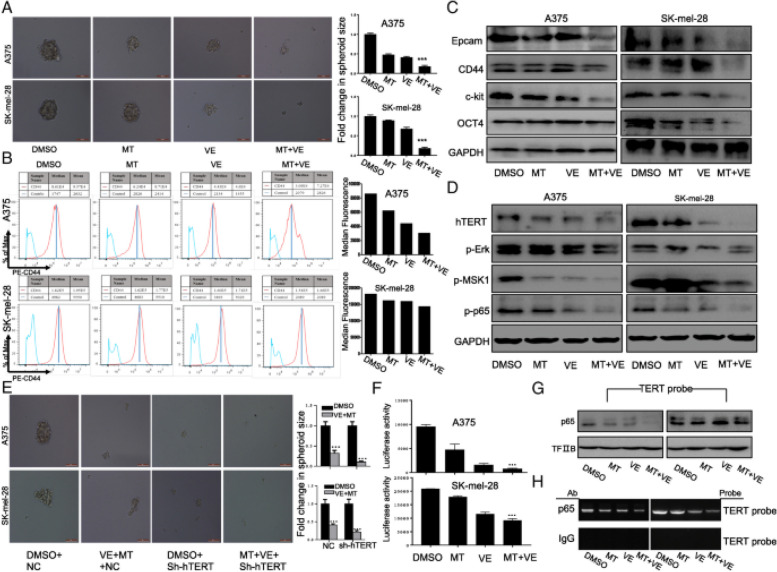
Combination of vemurafenib and melatonin inhibited cancer stem cell traits by down-regulating hTERT in melanoma cells. Human melanoma cells were exposed to vemurafenib (VE) (2.5 μM) with or without melatonin (MT) (1.0 mM) for 48 h. (**a**). The representative images of tumor sphere formation of melanoma cells with indicated treatment. (**b**). CD44 expression on the surface of melanoma cells was analyzed by FACS. (**c**). The expression of CSC-related markers Epcam, CD44, c-kit and Oct4 were determined by western blot in A375 and SK-mel-28 cells with the indicated treatment. (**d**). The expression of hTERT-p-MSK1-p65 pathway were determined by western blot in A375 and SK-mel-28 cells with the indicated treatment. (**e**). The representative images of tumor sphere formation of melanoma cells treated with DMSO or vemurafenib (2.5 μM) combined with MT (1.0 mM) for 24 h after pretreatment with the hTERT targeting shRNA. (**f**). Melanoma cells were co-treated with the plasmids of hTERT promoter driven-luciferase and vemurafenib (VE) (2.5 μM) with or without melatonin (MT) for 48 h followed by a dual-luciferase assay. The relative luciferase intensity per mg protein was calculated in the treated cells. (**g**). The streptavidin–biotin pulldown assay was performed to analyze the binding of P65 protein to hTERT promoter in melanoma cells with the indicated treatment. (**h**). Binding of p65 to the hTERT promoter in chromatin structure by ChIP assay. IgG, a negative control for ChIP in melanoma cells with the indicated treatment. The data are presented as the mean ± SD of three separate experiments. * *P* < 0.05, significant differences between treatment groups and DMSO control groups

**Correct Figure 5**

**Fig. 5:**
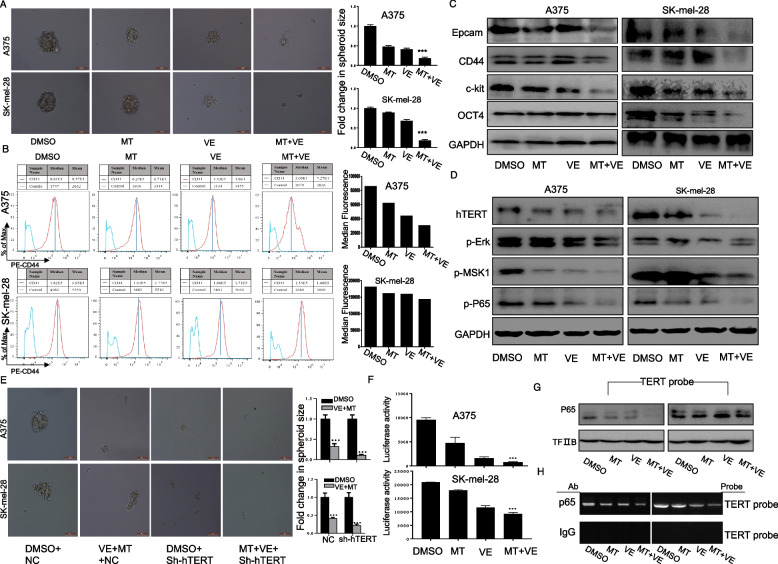
Combination of vemurafenib and melatonin inhibited cancer stem cell traits by down-regulating hTERT in melanoma cells. Human melanoma cells were exposed to vemurafenib (VE) (2.5 μM) with or without melatonin (MT) (1.0 mM) for 48 h. (**a**). The representative images of tumor sphere formation of melanoma cells with indicated treatment. (**b**). CD44 expression on the surface of melanoma cells was analyzed by FACS. (**c**). The expression of CSC-related markers Epcam, CD44, c-kit and Oct4 were determined by western blot in A375 and SK-mel-28 cells with the indicated treatment. (**d**). The expression of hTERT-p-MSK1-p65 pathway were determined by western blot in A375 and SK-mel-28 cells with the indicated treatment. (**e**). The representative images of tumor sphere formation of melanoma cells treated with DMSO or vemurafenib (2.5 μM) combined with MT (1.0 mM) for 24 h after pretreatment with the hTERT targeting shRNA. (**f**). Melanoma cells were co-treated with the plasmids of hTERT promoter driven-luciferase and vemurafenib (VE) (2.5 μM) with or without melatonin (MT) for 48 h followed by a dual-luciferase assay. The relative luciferase intensity per mg protein was calculated in the treated cells. (**g**). The streptavidin–biotin pulldown assay was performed to analyze the binding of P65 protein to hTERT promoter in melanoma cells with the indicated treatment. (**h**). Binding of p65 to the hTERT promoter in chromatin structure by ChIP assay. IgG, a negative control for ChIP in melanoma cells with the indicated treatment. The data are presented as the mean ± SD of three separate experiments. * *P* < 0.05, significant differences between treatment groups and DMSO control groups
