# Supplementary figures and images for: Melatonin synergizes BRAF-targeting agent vemurafenib in melanoma treatment by inhibiting iNOS/hTERT signaling and cancer-stem cell traits
Source: J Exp Clin Cancer Res. 2019 Feb 4;38:48. doi: 10.1186/s13046-019-1036-z (PMC6360719; doi:10.1186/s13046-019-1036-z)

A

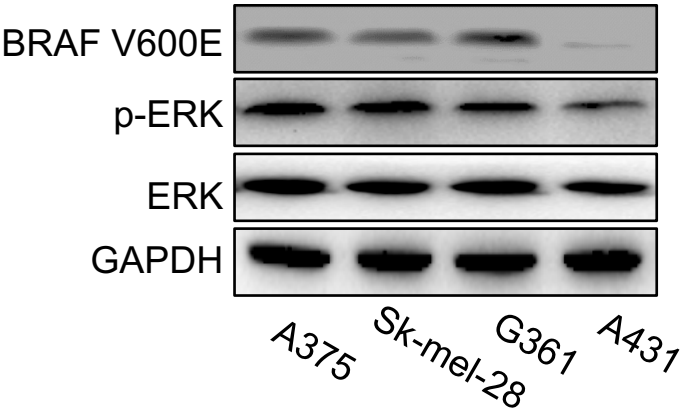

B

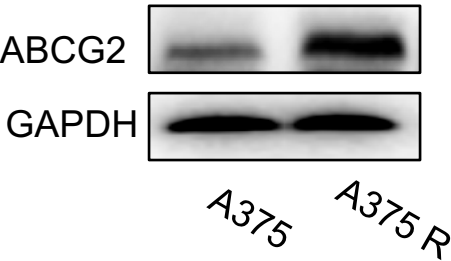

C

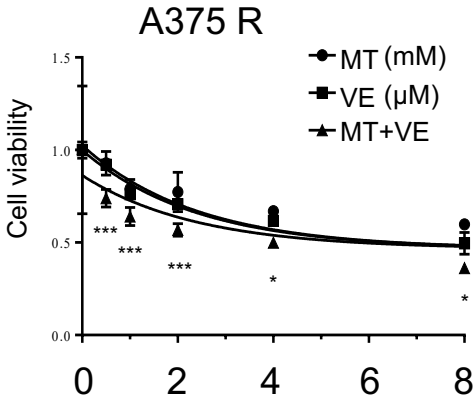

D

| Cell   | IC50     |         |            | Folds |
|--------|----------|---------|------------|-------|
|        | MT(mM)   | VE(μM)  | MT(1mM)+VE |       |
| A375 R | 14. 2049 | 7. 6332 | 2. 6223    | 2.91  |

A

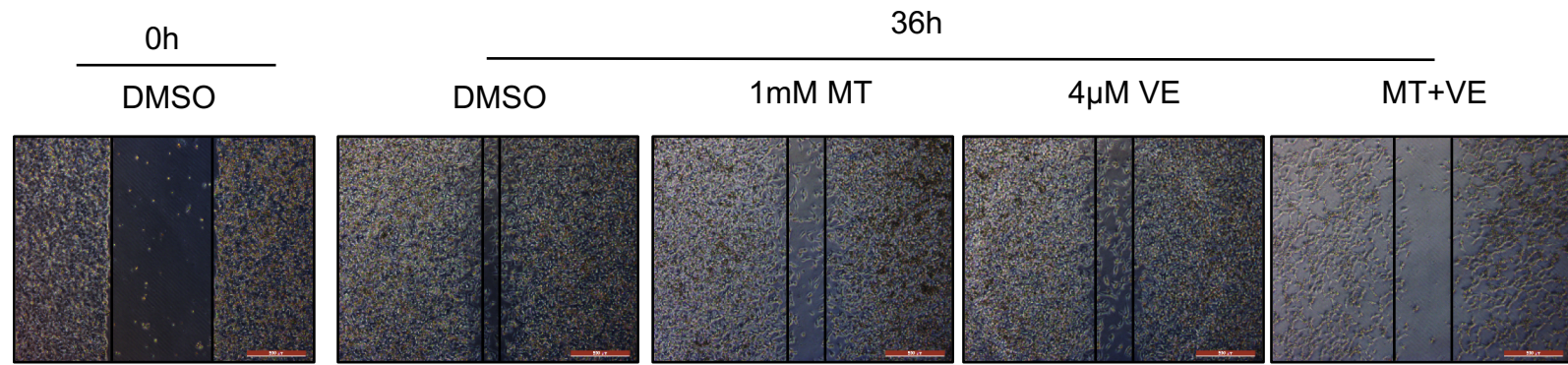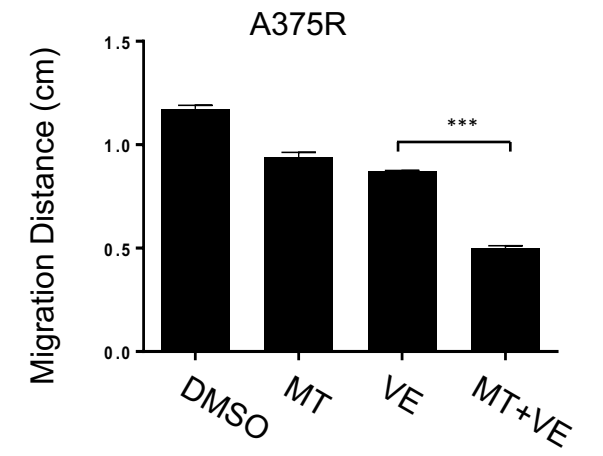

B

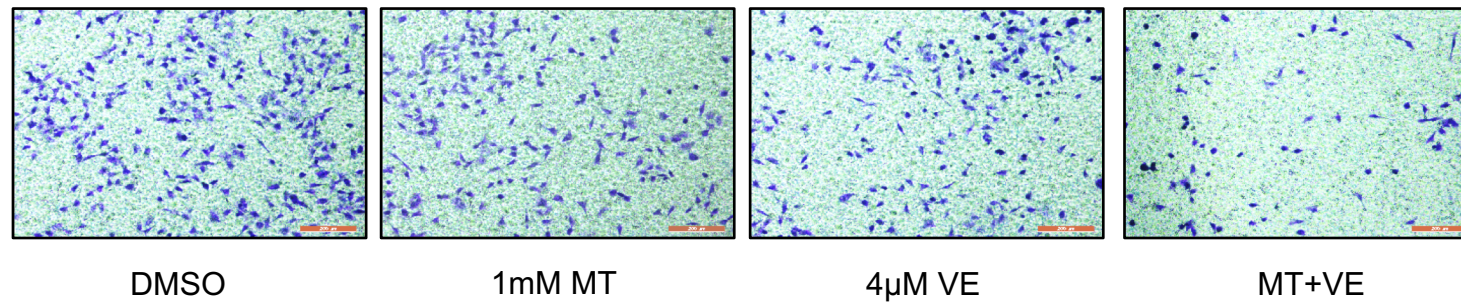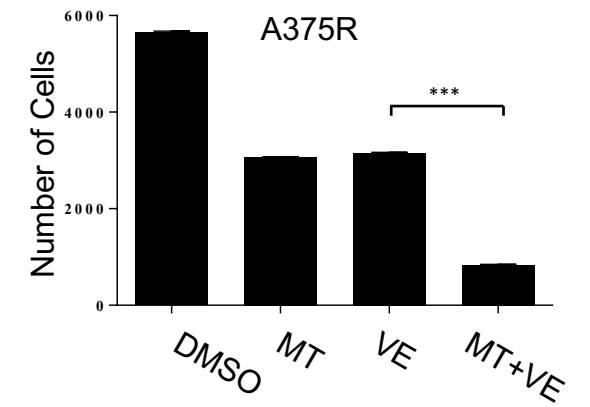

Supplement: Supplementary file 1 — Figure S1. Melatonin enhanced the inhibition of cell proliferation by vemurafenib. (A). BRAF V600E, p-ERK and ERK was respectively detected by Western blot assay in melanoma cells (A375, SK-mel-28, G361 and A431). (B). ABCG2 was respectively detected by Western blot assay in A375 and A375R cells. (C). Human melanoma cells (A375R) were treated with the increasing doses of vemurafenib (VE), melatonin (MT) alone or their combination for 48 h, and the cell viability was examined by MTT assay. (D). The IC50 values of vemurafenib (VE) for cell viability inhibition in A375R cells treated with or without melatonin (MT) were determined. Figure S2. Melatonin enhanced the inhibition of cell migration and invasion by vemurafenib (A). Cell migration was analyzed by a scratch assay. A375R cells were treated with vemurafenib (VE) (4 μM), melatonin (MT) (1.0 mM) or their combination. After 36 h, the wound gap was observed and photographed, and the distance of migration cells were calculated relative to the original gap. (B). Cell invasion was analyzed by a transwell assay in A375R cells with different treatment. The invaded cells were stained and observed, and the number of the invasion cells was presented. The data is presented as mean ± SD of three separate experiments, *P < 0.05, **P < 0.01, significant differences compared to the control groups. (PDF 4326 kb) [file 13046_2019_1036_MOESM1_ESM.pdf]
